# Research Progress on Intelligent Prediction, Debittering Technologies, and Multi-Dimensional Evaluation for Bitter Peptides

**DOI:** 10.3390/foods15132301

**Published:** 2026-06-27

**Authors:** Jun-Tong Wang, Cheng Luo, Cai-Xia Jiang, Xi-Qun Zheng

**Affiliations:** 1 College of Food Science, Heilongjiang Bayi Agricultural University, Daqing 163319, China; wangjuntong1248@163.com (J.-T.W.); luocheng07102001@163.com (C.L.); 2 Daqing Center of Inspection and Testing for Agricultural Products Ministry of Agriculture, Daqing 163319, China; 3 Key Laboratory of Agro-products Processing and Quality Safety of Heilongjiang Province, Daqing 163319, China; 4 National Coarse Cereals Engineering Research Center, Heilongjiang Bayi Agricultural University, Daqing 163319, China; 5 Engineering Research Center of Processing and Utilization of Grain By-Products, Ministry of Education, Daqing 163319, China

**Keywords:** food protein hydrolysates, molecular docking, deep learning, TAS2R receptors, electronic tongue

## Abstract

Bioactive peptides have health benefits, but the intense bitterness associated with their hydrolysis severely restricts their industrial applications. This paper systematically constructs a collaborative theoretical framework that integrates intelligent prediction, targeted debittering, and multi-dimensional evaluation. Firstly, it reviews the core applications of deep learning (such as quantitative structure–activity relationship (QSAR) and graph convolutional network (GCN)) combined with molecular docking technology in the high-throughput identification of bitter peptides and the analysis of target receptor interaction mechanisms. Secondly, it discusses how artificial intelligence and computational simulation can improve the efficiency of traditional debittering processes, emphasizing the advantages of multifunctional composite wall materials in the targeted encapsulation and delivery of bitter peptides, as well as the metabolic regulatory mechanisms behind controlling microbial fermentation for the debittering of specific peptide substrates. Finally, to provide a high-fidelity data closed loop for artificial intelligence (AI) models, a three-dimensional cross-validation system integrating standardized quantitative sensory evaluation and biomimetic electronic tongues was established. Future research should focus on developing large models for flavor generation to drive the green and targeted creation of low-bitterness and highly active peptides.

## 1. Introduction

The bioactive peptides produced by the hydrolysis of food-derived proteins, due to their remarkable physiological regulatory functions and health-promoting effects, have become the core focus in the development of functional foods [1]. These peptides exhibit various physiological activities including antihypertensive, antioxidant, hypoglycemic, immunomodulatory, and cholesterol-lowering effects. However, during the protein hydrolysis process, the natural spatial conformation is disrupted, causing a large number of hydrophobic amino acid residues that were originally enclosed within the molecule to be exposed. These hydrophobic regions interact with specific bitter receptors on the taste buds of the oral cavity, inevitably resulting in a strong bitter taste [2], which has become the main bottleneck restricting their large-scale industrial application [3,4]. Therefore, clarifying the intrinsic relationship between peptide structure and bitterness has become a key issue that urgently needs to be addressed in this research field [5]. The research has found that bitter peptides are widely present in dairy products, plant sources, and protein hydrolysates from animal by-products. Due to the exposure of hydrophobic groups after these substrates are hydrolyzed, they bind to oral taste receptors and trigger the bitter signal transduction pathway [6]. For instance, in the newly identified peptide sequences from soy protein hydrolysates, 22 high-molecular-weight components have been confirmed to be potential bitter peptides [7], and the bitterness induction of these polypeptides is closely related to their specific amino acid composition and sequence characteristics [8]. Although the academic and industrial communities have developed various debittering strategies such as physical adsorption, masking, microcapsule encapsulation, specific enzymatic hydrolysis, and microbial fermentation, the traditional processes often lack precision and are highly dependent on experience. For the embedding-based desensitization strategy, the core objective is to achieve phased control. At the oral stage, the aim is to prevent contact between the bitter peptide sequence and the TAS2R bitter receptor. At the gastrointestinal digestion stage, it allows the release to exert nutritional or physiological functions [9]. Therefore, a traditional masking and encapsulation system will result in insufficient targeted masking at the oral stage and inaccurate control of the release timing.

In recent years, the deep integration of artificial intelligence (AI) and big data technology with food processing and omics analysis has been revolutionizing the traditional paradigm of food flavor research. Intelligent algorithms have been applied in various scenarios of bitter peptide research. Taking complex protein hydrolysates such as soybean or sesame protein hydrolysates as examples, intelligent models can process large-scale peptide sequence data generated by liquid chromatography–tandem mass spectrometry (LC-MS/MS) and prioritize candidate bitter sequences, thereby reducing the dependence on exhaustive purification while still requiring fraction-level sensory validation [10]. By constructing deep learning architectures such as graph neural networks (GNNs), researchers can not only efficiently screen bitter peptides from proteomics data but also reveal the interaction mechanism between them and receptors. Combined with molecular docking and molecular dynamics simulations, the three-dimensional binding conformation of bitter peptides and human bitter receptors can be intuitively reconstructed at the atomic level [11]. After clarifying the precise structure of the bitter peptide and its receptor target, the subsequent debittering strategies will be more targeted: whether it is selectively removing the core bitter components, directing the screening of microbial strains that efficiently metabolize specific sequences, or reasonably designing multifunctional composite wall materials for precise masking of the taste, all can significantly reduce the research and development costs. This targeted approach provides strong technical support for achieving the “flavor and efficacy win–win” of active peptide products. At the same time, sensory evaluation and biomimetic electronic tongue technology play crucial roles in the verification of the closed-loop data system. This paper aims to construct a “smart prediction–targeted debittering–multi-dimensional evaluation” collaborative theoretical framework, providing scientific basis and methodological guidance for the bitter regulation of bioactive peptides, and thereby promoting their wide application in functional foods and special medical purpose formula foods.

## 2. Intelligent Prediction of Bitter Peptides

**Figure 1:**
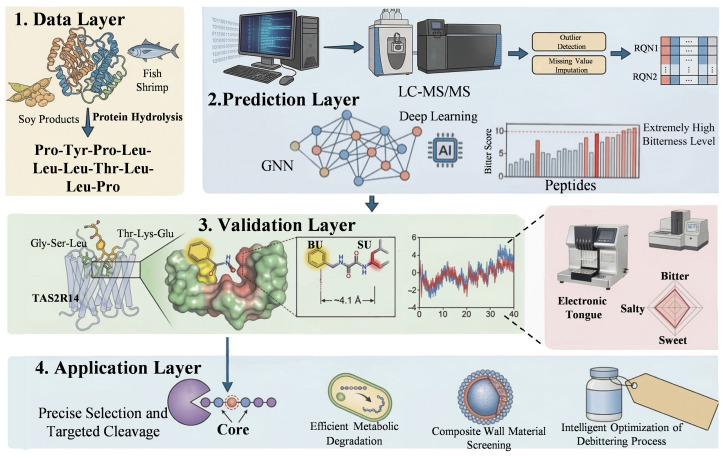
Hierarchical positioning of AI technology in the research of bitter peptides. Colors distinguish different receptor subtypes, arrows indicate the sequential workflow, and dashed lines denote detailed expansions or critical thresholds.

With their outstanding data processing capabilities, artificial intelligence and machine learning technologies have opened up a brand-new path for the research on bitter peptides. As shown in Figure 1, the four-level technical framework consisting of the data layer, prediction layer, verification layer, and application layer forms a logically rigorous progressive structure; among them, the continuous data flow and feedback mechanism establish a collaborative system, jointly driving the intelligent evolution of this research field.

Specifically, the data layer serves as the foundation of the entire framework, responsible for integrating multi-source heterogeneous data and extracting core features. The prediction layer focuses on the precise identification of bitter peptides and the quantitative modeling of their bitterness intensity. The verification layer’s role lies in conducting rigorous experimental verification of the prediction results through sensory evaluation and biomimetic sensing technologies (such as electronic tongues and electronic noses) [12]. Finally, for the application layer, as the terminal for the transformation of technological achievements, its core task is to achieve intelligent optimization of the debittering process. By using embedding and fermentation strategies to prepare low-bitterness peptides, this layer aims to provide targeted and efficient debittering solutions for the food industry [13,14].

### 2.1. Computational Prediction Based on the Q-Rule

The Q-rule proposed by Ney [15] is recognized as the first systematic theoretical model in the field of bitter peptide prediction. Its core principle lies in evaluating the potential taste properties of the peptide chain through the average hydrophobicity parameter (Q value). This model calculates the Q value based on the amino acid primary sequence and specific hydrophobic constants of side chains (see Equation (1)), thereby establishing a quantitative prediction benchmark. When the Q value exceeds 1400 cal/mol, the peptide segment exhibits a strong tendency towards bitterness. Although more complex models have emerged with technological advancements, as the foundational work, the Q rule still holds significant reference value in the preliminary screening of functional peptides and the regulation of food flavor to this day.

(1) $$Q=\frac{\sum \Delta g_{i}}{n}$$

where *Q* is the average hydrophobicity (cal/mol), $\Delta g_{i}$ is the transfer free energy of the *i*th residue, and *n* is the peptide length.

Previous studies have investigated the types and distribution of key hydrophobic residues in bitter peptides. Fu et al. [16] used the self-digestion product of shrimp heads as the research model and systematically analyzed the correlation between the intensity of the bitter taste and the hydrophobic parameters of the proteins. By constructing a synergistic hydrolysis system using exogenous flavor enzymes (Flavourzyme) and endogenous enzymes from shrimp heads, they found that the core bitter components in the hydrolysis products were mainly concentrated in the peptide segments of 3–5 kDa. Among the different molecular weight components separated, the components with a lower average hydrophobic value actually showed stronger bitterness. Moreover, the positioning of the hydrophobic amino acid residues at the N-terminal or C-terminal of the peptide chain determines their bitter properties, and the bitter response caused by the C-terminal residues is significantly stronger than that of the N-terminal residues. Song et al. [17] used Sephadex G-25 gel filtration chromatography to separate and purify 14 kinds of bitter peptides from yak milk hard cheese. Sequence analysis indicated that proline (Pro), valine (Val), leucine (Leu), isoleucine (Ile), and phenylalanine (Phe) were the most important hydrophobic amino acid residues in these bitter peptides. These specific hydrophobic amino acids constitute the core molecular basis of bitter peptides, and their presence directly determines the generation of the bitterness of the polypeptides.

### 2.2. Quantitative Structure–Activity Relationship (QSAR) Modeling

Quantitative Structure–Activity Relationship (QSAR) models integrate statistical and machine learning algorithms to establish a prediction system based on peptide sequences and structure descriptors. This modeling system was first proposed by Corwin Hansch and was initially applied in his pioneering research on benzoic acid derivatives [18]. Because QSAR models can identify multiple physicochemical properties as core predictive factors, this method has been widely recognized in related fields [19]. Therefore, a large number of researchers have used QSAR models to conduct peptide bitterness prediction, which not only deepens the basic research on the bitterness mechanism but also provides a solid theoretical basis for the intelligent evaluation of food flavor.

#### 2.2.1. Molecular Weight Prediction

The molecular weight of bitter peptides is generally concentrated between 500 and 1000 Da [20]. Studies have shown that peptide segments within this molecular weight range are the main source of the bitter taste in protein hydrolysates and contribute significantly to the overall flavor. From a mechanistic perspective, this is mainly because peptides within this size range have the optimal spatial conformation, which enables them to achieve precise spatial matching and efficient binding with the bitter receptor binding sites.

#### 2.2.2. Hydrophobicity Prediction

Hydrophobicity is one of the major physicochemical determinants of peptide bitterness, particularly for short peptides containing exposed aliphatic or aromatic residues. The proportion and exposure of hydrophobic residues are often used as important variables in bitterness prediction, but their contribution depends strongly on residue type, terminal position, neighboring residues, and peptide conformation. Moreover, surface hydrophobicity is negatively correlated with the bitterness threshold. The higher the surface hydrophobicity, the lower the bitterness threshold, indicating that the bitterness characteristics of polypeptides are more easily perceived by the senses [21]. Harmon et al. [22] systematically determined the intensity of bitter taste of free amino acids in 2024 and pointed out that Trp, Phe, Ile, Leu, Val, etc. are the most worthy of attention for strong bitter taste amino acids, while Arg also has obvious bitterness; Kohl et al. [23] proved from the receptor level that amino acids and peptide segments can activate multiple human bitter taste receptors.

However, in food systems, hydrophobic residues should not be simply regarded as “bitterness-inducing factors.” Some highly hydrophobic amino acids are also key components that determine the nutritional quality and functional value of proteins. Take branched-chain amino acids (BCAAs), which mainly include leucine (Leu), isoleucine (Ile), and valine (Val), for example. In recent years, they have been widely used in sports nutrition products and functional food formulations [22]. Among them, Leu is typically closely associated with signaling pathways related to muscle protein synthesis (MPS). Therefore, BCAAs are frequently incorporated into nutritional formulations aimed at balancing muscle protein synthesis and muscle protein breakdown (MPB) to enhance the protein utilization efficiency of sports nutrition foods [24].

Recent studies have even proposed a BCAA scoring system from an industrial food formulation perspective, designed to evaluate the potential contribution of protein or peptide components in sports foods toward nutritional efficiency related to muscle protein synthesis [25]. This indicates that Leu, Ile, and Val should not merely be viewed as hydrophobic residues linked to bitterness but should also be integrated into comprehensive evaluation systems for protein nutritional quality, functional retention, and food formulation optimization.

The research on the debittering effect of aminopeptidase demonstrated that the Leucine Aminopeptidase A (LapA) derived from Aspergillus oryzae primarily cleaves the hydrophobic amino acids at the N-terminus of the protein hydrolysate, including Leu, Val, Ile, and Phe, while releasing some other amino acids. After treatment, the content of these free hydrophobic amino acids in the hydrolysate significantly increased, but the overall bitterness of the system did not increase as a result; instead, the bitterness score of the hydrolysate decreased from 2.9 to 0.54 [26]. This indicates that the reduction in bitterness is not caused by a decrease in the concentration of free amino acids but rather by the breakage of peptide chains, which disrupts the original conformation of the peptide segments and damages the optimal structure of the bitter ligands, thereby weakening the stimulation of the taste receptors. Therefore, for bitter peptides, the exposure state of the terminal hydrophobic residues and the sequence environment formed with adjacent residues are more crucial in determining the actual intensity of bitterness than the absolute concentration of free hydrophobic amino acids. To avoid equating the number of hydrophobic residues with bitterness intensity, Table 1 summarizes the bitterness characteristics of common amino acid residues in free amino acid systems and their potential roles in bitter peptides. This residue-level comparison highlights why hydrophobicity-based prediction should be interpreted together with residue type, terminal exposure, sequence context, and receptor accessibility.

Therefore, hydrophobicity should be used as a risk indicator rather than as the only criterion for bitterness prediction or debittering design. Non-selective adsorption, membrane separation, or excessive enzymatic hydrolysis may reduce bitterness by removing hydrophobic peptides, but they may also eliminate BCAA-rich or bioactive sequences. A more appropriate strategy is to distinguish sensory-relevant bitter motifs from nutritionally valuable hydrophobic peptides by integrating peptide abundance, sensory thresholds, predicted TAS2R-binding risk, and known bioactive motifs. High-bitterness and low-functionality sequences can be selectively removed or blocked, whereas nutritionally valuable BCAA-rich peptides should preferably be retained and managed through oral masking, encapsulation, controlled release, or receptor-blocking strategies.

#### 2.2.3. Specificity of Amino Acid Sequences

The sequence distribution of amino acids, especially at the N- and C-termini, strongly influences peptide bitterness. In dipeptides, C-terminal hydrophobic residues often contribute substantially to bitterness perception, whereas N-terminal hydrophobic residues and C-terminal basic residues may further enhance bitterness. For tripeptides and tetrapeptides, bitterness is also affected by terminal hydrophobicity, side-chain volume, and the electronic properties of neighboring residues [27,28]. Taking the β-casein octapeptide (RGPFPIIV) as an example, its bitter threshold is as low as 0.004 mM, presenting a sensory intensity 250 times that of caffeine. However, when this exact sequence is completely reversed to VIIPFPGR, its bitterness weakens significantly, with the threshold rising to 0.14 mM [28]. This strongly demonstrates the core position of sequence arrangement in determining bitterness. Furthermore, the impact of sequence length and specific residues can be observed through deletion comparisons. For instance, the heptapeptide RGPFPIV (derived by omitting an isoleucine) exhibits a threshold of 0.11 mM, which is comparable to its reversed sequence VIPFPGR (threshold 0.07 mM). Deeper residue deletion experiments further highlight the role of terminal amino acids. When the N-terminal arginine (Arg) and glycine (Gly) are removed from the original octapeptide (RGPFPIIV) to generate the hexapeptide PFPIIV, the bitterness threshold increases to 0.13 mM. This confirms the key contribution of the N-terminal residues in maintaining the extremely high bitterness intensity of the original octapeptide.

In addition, circular dichroism (CD) analysis reveals a deeper mechanism. RGPFPIV and VIPFPGR, which have similar bitterness intensities, have highly similar secondary structure characteristics [29]. It can be seen that the bitterness of peptides is not solely determined by the primary sequence but is also profoundly influenced by their spatial conformation. Molecular weight, hydrophobicity, and terminal sequence specificity constitute the three key physical and chemical dimensions that determine the intensity of the bitterness of peptides. Through the quantitative analysis of these properties, QSAR models provide an indispensable theoretical basis for elucidating the molecular mechanism of peptide bitterness.

Kim and Li-Chan [30] conducted an in-depth analysis of the correlation between the structure and properties of bitter peptides using QSAR models. The study found that when hydrophobic amino acids are located at the C-terminal or basic amino acids are located at the N-terminal, dipeptides tend to exhibit higher bitterness intensity. Considering that model construction relies on the existing bitterness threshold database, Xu and Chung [31] further integrated 14 amino acid descriptors and constructed prediction models including dipeptides, tripeptides, and tetrapeptides. The results confirmed that the hydrophobic amino acids at the C-terminal are the primary factor determining the bitterness of dipeptides, followed by the large-volume hydrophobic residues at the N-terminal. Taken together, these QSAR studies indicate that peptide bitterness is primarily associated with intrinsic molecular descriptors, including peptide length, hydrophobicity, terminal residues, charge distribution, and sequence-dependent structural features. However, this does not mean that bitterness is completely independent of peptide source. In real food hydrolysates, the protein source and enzyme specificity determine which peptide sequences are released and at what abundance, while processing conditions and food-matrix interactions further influence whether these peptides remain accessible to TAS2R receptors and exceed sensory thresholds. Therefore, QSAR models should be regarded as first-line tools for estimating intrinsic bitterness potential, but their predictions require source-specific validation by peptidomics; sensory or electronic tongue analysis; molecular docking; and, where possible, receptor-based assays.

QSAR models are useful because they rapidly relate peptide descriptors, such as molecular weight, hydrophobicity, terminal residues, and charge distribution, to bitterness intensity. However, their predictive power depends strongly on the quality of threshold datasets, descriptor selection, and validation across different protein sources.

However, traditional QSAR models have obvious physical and chemical drawbacks when applied to complex food systems. The models overly rely on sequence and overall hydrophobic parameters but ignore the physical and chemical behaviors of peptide segments in aqueous food matrices or oral saliva environments. In a real aqueous solution, peptide chains do not present a rigid linear arrangement but exhibit high flexibility [32]. For instance, although some peptide chains have strong bitter-hydrophobic residues at their C-terminus, to minimize the surface energy of the system, peptide chains tend to spontaneously fold and coil. This process causes the bitter-hydrophobic groups to curl inward and be encapsulated within the molecular core, while exposing the hydrophilic groups on the outside for hydration.

Nowadays, through molecular docking technology, it can be seen that the effective binding of the polypeptide to the TAS2R14 receptor is highly dependent on a clear spatial conformation. The core binding sites usually require exposed Pro, Phe, and Trp side chains for precise anchoring, and the concealment or introduction of hydrophilic residues such as Gly and Glu will significantly weaken this binding stability due to the inability to form a stable spatial embedding. Because traditional QSAR models can only simply interpret the primary sequence information but cannot predict the conformational shielding effect that occurs in real solution systems, this limitation often leads to the model mistakenly classifying peptides without bitterness as high-bitter substances in sensory evaluation, resulting in a disconnection between the predicted results and the actual sensory experience [33,34].

### 2.3. Fractionation-Based Identification of Bitter Peptides

In bitter peptide studies, sensory-guided fractionation is essential for linking peptide sequences to actual bitterness. Complex hydrolysates are first divided into fractions by ultrafiltration, reversed-phase chromatography, multi-dimensional preparative LC, or crossflow filtration. Fractions with stronger bitterness or bitterness persistence are then prioritized for LC-MS/MS-based peptidomics and sequence elucidation [35,36].

This multi-dimensional process integrating fractionation, sensory evaluation, and peptidomics can significantly improve the reliability of bitter peptide identification and provide a more robust basis for subsequent debittering design and model validation. Liu et al. [35] identified bitter peptides in whey protein hydrolysate using sensory-guided fractionation combined with offline two-dimensional RP-HPLC, showing that chromatographic separation guided by bitterness evaluation can effectively narrow the search range for bitterness-contributing peptides. Daher et al. [36] combined trained sensory evaluation with RP-HPLC-HRMS/MS-based peptidomics and principal component analysis (PCA) to compare sixteen enzymatic hydrolysates produced from a milk protein liquid fraction enriched in micellar caseins and demonstrated that peptide profiles obtained by mass spectrometry could be statistically associated with sensory descriptors such as bitterness and bitterness persistence. In a subsequent sensopeptidomic kinetic study, the same group integrated sensory evaluation, time-resolved peptidomics, heat maps, decision trees, and random forests during micellar casein hydrolysis and identified 22 peptides that most strongly influenced bitterness [37]. Ongkowijoyo et al. [38] further applied offline multi-dimensional sensory-guided preparative LC fractionation to pea protein isolate and identified PA1b as a major bitter peptide; quantitative LC–MS/MS confirmed that its concentration reached 129.3 mg/L, exceeding the reported bitter sensory threshold of 3.8 mg/L. These studies indicate that multi-dimensional fractionation, sensory validation, and peptidomics are complementary rather than interchangeable steps in bitter peptide discovery. Therefore, computational prediction and molecular docking should be used after, or at least cross-validated by, fraction-level sensory evidence to avoid assigning bitterness to peptides that are detectable by MS but not sensorially relevant.

### 2.4. Data Screening Based on Peptidomics and Molecular Docking

After bitterness-relevant fractions and candidate peptide sequences are identified, receptor-level analysis is required to explain how these peptides interact with human bitter taste receptors. The perception of bitterness in humans is essentially a physiological defense mechanism, aimed at preventing the intake of potentially harmful substances. During food processing, the exposure of hydrophobic groups within peptide sequences accidentally activates this sense, resulting in the production of unpleasant bitterness in the product. The molecular basis of bitterness perception lies in the bitter receptor family (T2R) located on the surface of type II receptor cells in the taste buds of the tongue. The human genome encodes approximately 25 highly polymorphic T2R receptors [39], all belonging to the G protein-coupled receptor (GPCR) family, which is the largest membrane protein receptor class in the human body. These receptors have a typical seven-transmembrane helix (TM1 to TM7) structure. Among the numerous subtypes, T2R1, T2R4, T2R14, and T2R16 have been confirmed to be key targets for identifying food-derived bitter peptide [40]. Among these receptors, TAS2R14 is frequently used as a modeling target because of its broad ligand-recognition profile and its reported involvement in peptide bitterness recognition.

**Figure 2:**
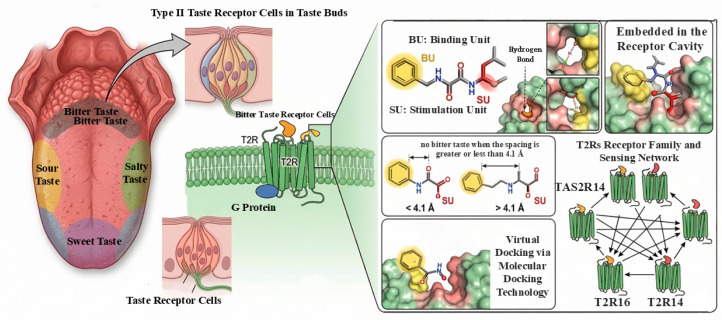
Peptide bitter taste perception and bitter taste signal transduction mechanism. Black arrows indicate the signal flow generated by bitter peptides in the T2R receptor family, forming the taste perception network.

As illustrated in Figure 2, the binding of bitter peptides to receptors follows an induced-fit dynamic model rather than simple rigid spatial matching. The bitter-inducing peptides usually contain a binding unit (BU) and a stimulating unit (SU), with a spatial distance of approximately 4.1 Å [41]. When the peptide ligand enters the extracellular binding pocket of the receptor, its hydrophobic side chain or specific charge of the stimulating unit will disrupt the hydrogen bond network and ionic bonds that maintain the receptor in a static state through steric hindrance and electrostatic interactions [42]. Subsequently, TM6 (the sixth transmembrane helix) undergoes an outward displacement, activating the intracellular binding domain of the receptor, thereby facilitating the insertion and coupling activation of the downstream G protein [43]. This series of dynamic structural changes converts the chemical signals of the polypeptide across the membrane into neural signals that can be recognized by the central nervous system, achieving a complete closed loop from oral tasting to brain taste perception. The elucidation of this transmembrane signal transduction mechanism not only reveals the fundamental cause of bitterness but also provides a solid theoretical basis for the subsequent intelligent screening of bitter peptides and the targeted debittering process in the food industry.

Peptidomics, usually based on LC-MS/MS, provides sequence-level information on peptides in complex hydrolysates and supplies candidate ligands for subsequent receptor-level analysis [44]. When combined with molecular docking, this approach can link peptide abundance and sequence features with predicted TAS2R interactions, thereby helping to prioritize peptides for sensory or receptor-based validation [45]. This technology effectively avoids the shortcomings of traditional prediction models in obtaining real amino acid sequences, thereby providing a highly accurate sequence basis for subsequent structural simulations. In addition to mining bioactive peptides, peptidomics is also essential for tracking bitter or off-taste peptides in complex food hydrolysates.

Recent studies have shown that by combining proteomics with molecular docking technology, low-bitterness antihypertensive peptides have been successfully identified from sesame proteins, and the molecular dynamics process of their formation with the key residues of the TAS2R14 bitter taste receptor has been predicted [10]. As a technology dedicated to comprehensive qualitative and quantitative analysis of all peptides in biological samples, proteomics highly relies on LC-MS/MS for high-throughput screening. Its core focus lies in analyzing the hydrophobicity, molecular weight, and terminal amino acid structure (such as HAA-BAA type) of peptides. By integrating mass spectrometry technology with statistical analysis, proteomics conducts an overall analysis of the entire peptide group and obtains precise peptide sequences from hydrolysis products. Coupling this method with molecular docking provides convenience for the screening of dual-functional peptides with both enhancing flavor and debittering properties, significantly expanding the boundaries of debittering processes.

Currently, proteomics has been widely applied to precisely track the characteristic bitter sequences in complex foods such as dairy products, aquatic products, and meat products. In the study of milk proteins, Hu et al. [46] utilized proteomics to dynamically detect the enzymatic hydrolysis process of casein, revealing the mutual influence of hydrolysis degree on digestibility and bitter release, providing a scientific basis for controlling the generation of bitter sequences. Kuhfeld et al. [47] combined cross-flow filtration with proteomics to systematically analyze the complete peptide profile of aged cheddar cheese, successfully identifying 5 peptides derived from β-casein, such as YPFPGP, as the core bitter substances, achieving efficient targeted identification of bitter peptides in complex matrices. In the field of fermented meat products, Dai et al. [48] utilized the powerful analytical capabilities of proteomics to precisely identify the key bitter peptides in dry-cured ham. Through LC-MS/MS, 11 core bitter peptides were intelligently identified and quantified, among which PKAPPAK, VTDTTR, and YIIE exhibited the most significant high bitter values.

Based on the precise ligand sequences provided by proteomics, molecular docking technology further elucidated the spatial interaction patterns between these peptides and broad-spectrum bitter receptors at the atomic level. Through computational simulation, researchers not only deciphered the mechanism of bitter production but also reverse-screened efficient competitive inhibitors [49,50]. Wei et al. [51] first discovered AP-6 and LT-5 peptides in yellowfin tuna that have both enhancing flavor and masking bitterness functions. By confirming their competitive inhibitory effect in the binding pocket of TAS2R14, it was confirmed that they have a strong debittering potential. Yu et al. [52] screened peptides with inhibitory activity against TAS2R14 from rainbow trout proteins. Molecular docking confirmed that these peptide segments could occupy the active pocket of the receptor through specific hydrogen bonds and hydrophobic networks, effectively blocking the transmission of bitter signals. This “omics-computation” collaborative strategy significantly broadened the research field of bitter removal in food processing. Subsequently, the results were verified through in vitro electronic tongue tests. These studies fully demonstrated the long-term application value of proteomics and molecular docking technology in the efficient screening of functional peptides. Thus, a complete research workflow was established.

First, proteomics was used to extract target peptide sequences from complex hydrolysis products; then, machine learning and molecular docking techniques were employed for high-throughput virtual screening and receptor mechanism analysis; and finally, their actual flavor regulation function was verified through sensory evaluation or a biomimetic electronic tongue. Although the molecular dynamics trajectories provided by computational chemistry can precisely depict the influence of minor conformational changes on taste characteristics, considering the inherent conformational flexibility of ligands and the accuracy limitations of existing scoring systems, multi-dimensional cross-validation of the extracellular environment is still an indispensable core link in this closed system.

### 2.5. Deep Learning Models

Although proteomics has successfully linked sensory data to bitterness thresholds and receptor activity, this technology still faces significant challenges. The detection limit of mass spectrometry signal intensity often reduces the sensitivity of detecting specific peptides. In this context, machine learning (ML) models, with their ability to precisely capture complex molecular internal relationships, provide a feasible technical solution to break through this limitation [53].

The early prediction methods were highly dependent on one-dimensional sequence information, while modern artificial intelligence technologies have demonstrated significant advantages in analyzing three-dimensional spatial effects [54]. In this trend, graph neural networks (GNNs) have become a research hotspot in the academic community because they can abstract polypeptide molecules into topological graph structures, thereby directly mapping and describing the complex nonlinear relationships between different amino acid residues [55]. As the first application case of graph convolutional networks (GCNs) in the prediction of bitter peptides, the BitterPep-GCN model constructed by Srivastava et al. [11] successfully broke through the dimension limitations of traditional sequence models. To further expand the broad potential of graph neural networks in the feature extraction of polypeptides, Chen et al. [56] subsequently developed a new lightweight graph deep learning framework called TP-LMMSG. This framework flexibly integrates the heterogeneous features and spatial connectivity of polypeptides, demonstrating excellent generalization ability in the prediction of various bioactive peptides, laying a key methodological foundation for the high-throughput targeted screening of bitter peptides.

Based on this, as the specific application of GCNs in bitter analysis, the BitterPep-GCN model successfully broke through the dimension limitations of traditional sequences. By combining amino acid embedding representations and mixed pooling strategies, it not only accurately identified key bitter residues (such as F, G, P, and R) but also further clarified the specific sequence motifs that determine the intensity of bitterness [11]. Its outstanding classification accuracy and ROC-AUC performance not only provide an efficient virtual screening tool for food debittering but also offer a new perspective for decoding the bitterness mechanism from the spatial topological perspective.

In recent years, the development of large-scale pre-trained models has further shaped the research model in bioinformatics. The BIPE (Bitterness Intensity Prediction Engine) system utilizes a large-scale protein language model and, by absorbing the sequence semantic information from a vast dataset of food protein hydrolysates, conducts quantitative regression on the bitterness threshold within a logarithmic space. Unlike traditional trial-and-error methods, advanced computational platforms can more accurately capture potential bitterness features. For instance, the iBitter-GRE model integrates the ESM-2 protein language model with multi-view features to predict bitterness threshold regression [57], while the BitterEN model leverages a novel ensemble learning framework to significantly improve the high-throughput identification accuracy of bitter peptides [58]. For peptides with a molecular weight below 3000 Da, such computational assistance platforms can precisely locate their high-bitterness risk sequences. This provides highly efficient virtual screening tools for food researchers, allowing them to eliminate high-bitterness sequences and optimize targeted enzymatic digestion processes at an early stage.

However, for these machine learning models to gain genuine recognition in food engineering, a complete data loop must be formed through rigorous validation. To address this issue, Yu et al. [59] developed a CPM-BP prediction model driven by a lightweight gradient boosting algorithm and constructed a comprehensive debittering process integrating proteomics, machine learning, and cytological experiments. After identifying 724 peptide segment differences between spoiled milk and fresh milk, the CPM-BP model successfully screened out 180 sequences with potential bitterness. Subsequently, the research team conducted in vitro physiological tests using an engineered HEK293T cell line that stably expresses the human T2R4 taste receptor. The experiments confirmed that the targeted sequences predicted by the model, especially FALPQYL and FFVAPFPEVFGKE, successfully activated the receptor and initiated the bitter taste signaling pathway. By directly linking the underlying computational predictions with the physiological responses at the cellular level, this study strongly demonstrated that the AI model has the practical ability to guide the real food matrix debittering process.

Although there are already a large number of high-precision deep learning models available, blindly applying the latest algorithms is not the optimal solution in the food debittering process. The hydrolysis products of different protein matrices vary significantly in terms of peptide chain length, conformational flexibility, and modification characteristics. This inherent heterogeneity can cause significant fluctuations in the generalization ability of the model and the practical application boundaries. To solve this problem, we have constructed a multi-dimensional decision matrix (Table 2) specifically for the intelligent bitter peptide prediction model. This model comprehensively considers the physical and chemical differences between the matrix and experimental conditions, aiming to provide a scientifically rigorous decision-making basis for researchers in selecting models.

Although artificial intelligence and deep learning have shown great potential in the virtual screening of bitter peptides, blindly believing in the prediction results of a single model often leads to misleading conclusions. Once these computational tools are applied to real food systems, their deep limitations in physical and chemical modeling and data generalization capabilities will become apparent.

From the perspective of molecular structure, whether it is sequence-based regression analysis or spatial docking simulation, the existing algorithms are unable to accurately reproduce the dynamic three-dimensional folding process of peptides in complex aqueous solution environments. Moreover, the real food matrix is rich in macromolecules such as lipids and polysaccharides, which have a significant physical masking effect on the release of bitterness. These macromolecular-level interferences are often overlooked by the computational models. Due to the failure to consider the physical steric hindrance in these real systems, the predicted results output by the algorithms often have a serious deviation from the actual human sensory evaluation.

Another core constraint stems from the limitations of the training data itself. Even the most advanced large models are limited by the quality of the underlying proteomics datasets in terms of their prediction accuracy. Since most current deep learning tools are trained based on highly specific protein sources, their reliability decreases when faced with unfamiliar biological matrices. Especially when the models are required to analyze highly complex mixed food systems, emerging insect proteins, or ultra-long peptide chains produced during fermentation, their recognition capabilities often significantly decline. How to overcome the lack of stability in cross-matrix prediction is a key bottleneck that this field needs to address in the future.

## 3. AI-Targeted Debittering of Bitter Peptides

Bitter peptides are widely present in almost all protein hydrolysates of food. Due to the fact that their formation pattern and structural profile are highly dependent on the protein composition of the raw materials, the physicochemical behaviors of these peptides vary in different food matrices. Therefore, industrial bitterness removal cannot rely on a simple scheme. For different products, the specific physicochemical properties of bitter peptides and the technical barriers to their removal vary significantly.

For such bitter off-taste problems, the industrial sector currently relies mainly on physical methods, enzymatic hydrolysis, or biological fermentation. The core mechanism of physical intervention techniques (including activated carbon adsorption, microcapsule encapsulation, and masking) lies in spatially isolating the bitter compounds or directly blocking the bitter taste perception in the mouth. Enzymatic debittering involves introducing specific enzyme preparations to cut the target sequences that trigger the bitter taste. The biological method mainly relies on microbial fermentation, using the continuously secreted peptidases of living microorganisms to target and degrade the bitter polypeptides.

In recent years, driven by intelligent algorithms and omics data, the debittering process has been transforming towards more refined strategies. Researchers utilize intelligent detection technologies to precisely identify the core characteristics of bitter molecules, thereby scientifically integrating traditional debittering methods and effectively avoiding the previous trial-and-error approach. This data-driven new path has reshaped the debittering process, not only achieving efficient removal of undesirable bitterness but also strictly preserving the potential physiological effects of bioactive peptides.

### 3.1. In Silico Physical Debittering

The debittering efficiency of traditional physical adsorption or masking techniques is highly susceptible to interference from other substances in food and is largely limited by the empirical and blind trial-and-error approach. In recent years, the introduction of molecular simulation technology has endowed physical debittering and masking agent screening with the ability of computer virtual prediction. By constructing the three-dimensional spatial conformation of bitter peptides, researchers can use the computational model to precisely quantify the binding free energy between the target peptide segment and specific receptors, separation media, or masking molecules.

Furthermore, the taste peptide docking machine (TPDM), as a machine learning-assisted taste peptide prediction tool, provides a representative example of integrating docking-derived information with molecular descriptors and fingerprints for bitter peptide discrimination. Cong et al. [60] conducted research using TastePeptidesDM (TPDM) for bitter peptide prediction. They collected 18 tripeptides and tetrapeptides from Chinese liquor and conducted preliminary bitter prediction using the TastePeptidesDM 2024 version. This module returns a binary classification result of “bitter” or “no bitterness” and provides a confidence score for “bitterness”. Subsequently, the researchers verified the prediction results through a trained sensory panel and found that the peptide segment WIKK had the strongest bitterness. Further analysis of the subsequent bitter mechanism was conducted using the T2R47 receptor, cell calcium flow experiments, molecular docking, and molecular dynamics simulations.

#### 3.1.1. Flavor Masking

Flavor masking represents a classic non-destructive debittering strategy that introduces specific flavor compounds or polysaccharides to induce competitive inhibition or physical blockade at the taste bud receptor level [61]. Specific combinations of additives or polysaccharides are incorporated into protein hydrolysates to achieve important bitterness-masking effects [62]. Currently, various sweeteners, acidifiers, and flavorings have been proven to be effective inhibitors of bitterness and can weaken the sensory perception of bitterness [63]. Bertelsen et al. [64], based on the interaction mechanism of sweet and bitter tastes, systematically investigated the masking efficacy of xylitol and sucrose on enzymatically hydrolyzed soy protein (E-SP). The research results revealed a key issue: Although these two masking agents can significantly reduce bitterness in a single aqueous solution model, they are almost ineffective in real food matrices.

From this, it can be seen that the debittering effect of masking methods is highly susceptible to interference from complex food matrices, resulting in extremely unstable actual performance. This reveals the application shortcomings in real food systems, highlighting the challenges of achieving efficient debittering.

#### 3.1.2. Selective Separation

The selective separation technology mainly targets and removes bitter compounds from enzymatic hydrolysis products through means such as adsorption, solvent extraction, ultrafiltration, precipitation or chromatography [2,65]. Activated carbon adsorption is a commonly used physical approach for reducing bitterness in protein hydrolysates [66]. Its mechanism mainly relies on hydrophobic adsorption, supplemented by hydrogen bonds and electrostatic interactions, thereby preferentially adsorbing high-molecular-weight hydrophobic peptides rich in aromatic amino acids. Su et al. [67] applied this method to the debittering study of egg white protein hydrolysates; the results showed that by regulating the degree of hydrolysis, the effective molecular weight grading of peptide components could be achieved. After optimizing the pH value to regulate the adsorption efficiency, the removal rate of phenylalanine reached 82%, and the overall bitterness value was reduced to 70%. However, separation-based debittering is not inherently selective. Bitter peptides and bioactive peptides often share similar physicochemical features, including small size, high hydrophobicity, exposed aromatic or branched-chain residues, and strong affinity for adsorbents or membranes. Therefore, adsorption, ultrafiltration, precipitation, and chromatography may reduce bitterness but can also co-remove nutritionally valuable or bioactive peptides. This is particularly important for BCAA-rich sequences. A more rational strategy is to distinguish sensory-relevant bitter fractions from nutritionally valuable hydrophobic peptides by integrating peptide abundance, sensory thresholds, predicted TAS2R-binding risk, and known bioactive motifs. High-bitterness and low-functionality fractions may be selectively reduced, whereas valuable hydrophobic peptides should be retained and managed through masking, encapsulation, controlled release, or receptor-blocking strategies.

Molecular docking and molecular dynamics simulations provide a new solution based on the thermodynamic perspective to break through this technical barrier. When screening resins or adsorption materials, researchers no longer need to rely on blind experimental trial and error. Instead, by precisely calculating the stacking forces and electrostatic repulsive energies between the microscopic structure of the adsorbent surface and the hydrophobic groups exposed by the bitter peptide, the quantitative analysis of the adsorption behavior can be achieved. Moreover, computational simulations can accurately screen out adsorption media with pore diameters highly matching the hydration radius of the bitter peptide and whose polar microenvironment can specifically anchor the bitter-inducing motif. This virtual screening-to-targeted adsorption strategy utilizes steric hindrance and specific non-covalent interactions to precisely capture the bitter peptide while retaining the biological activity peptide.

#### 3.1.3. Solvent Extraction

The solvent extraction technique is based on the principle of polarity similarity and solubility and is an effective method for separating bitter peptides. However, this method often introduces the risk of organic reagent residue while significantly reducing the bitterness [68]. Under this methodology, Gupta et al. [69] used an ultrasonic-assisted solvent extraction method to debitterize grapefruit juice, confirming that the ultrasonic cavitation effect can significantly promote molecular diffusion and improve reaction kinetics. Although solvent extraction and ultrasound-assisted extraction have been used to reduce bitterness in some non-peptide food systems, their direct application to bitter peptide hydrolysates remains limited by solvent-residue risk, peptide loss, and possible activity reduction.

The debittering effect of traditional membrane separation technology often lacks certainty, and the core technical difficulty lies in the inability to achieve a precise match between the molecular weight cut-off (MWCO) and the membrane material. Moreover, due to the high conformational flexibility of peptides in aqueous environments, traditional two-dimensional molecular weight assessment methods often fail to yield effective results. Therefore, in the field of food chemistry, molecular dynamics (MD) simulation technology has been introduced to predict the surface charge distribution of specific bitter peptides in aqueous solutions, thereby screening out membrane materials with the most suitable pore diameters and hydrophilic/hydrophobic properties. This strategy not only effectively alleviates the adhesion and contamination problems of hydrophobic bitter peptides on the membrane surface but also significantly improves the selectivity and industrial operation efficiency of membrane separation for debittering, providing strong theoretical support for the scientific selection of membrane materials and process optimization.

### 3.2. AI-Driven Encapsulation Debittering Technology

By using encapsulation technology to construct a microscopic physical barrier to prevent the hydrophobic groups of bitter peptides from coming into contact with the TAS2R family receptors in the oral cavity, this is one of the most crucial strategies currently employed in the food industry [70]. Moreover, an ideal encapsulation system not only needs to physically shield the perception of bitterness through spatial steric hindrance effects but also should allow the peptide segments to be released controllably under gastric or intestinal conditions, thus ensuring the targeted release and functional performance of bioactive peptides [9]. However, in the face of highly complex food matrices and large molecule interface behaviors, the traditional empirical driven screening mode for wall materials often encounters severe bottlenecks. This often results in low encapsulation efficiency and poor controlled-release stability, which constitutes the core limitation of the traditional method and further indicates that conventional screening methods often cause the wall materials to fail in complex matrix systems.

#### 3.2.1. Molecular Inclusion and Gel Network Entrapment

In 1996, Willaert and Baron [71] demonstrated that the gel technology could retain cell viability to the greatest extent. However, although alginate and the enhanced molecular intercalation reaction can provide important structural support for the gel matrix, insufficient strength and limited stability remain the key bottlenecks it faces. In this context, using oligosaccharides with specific cavity structures for host guest recognition has become an efficient molecular level encapsulation strategy for low-molecular-weight and strongly hydrophobic bitter peptides [72,73], and this technology is also one of the important processes for improving food flavor in industrial production [74]. For example, Abbasi et al. [75] used cyclic molecules with a hydrophobic inner cavity and a hydrophilic outer surface as host molecules. By leveraging van der Waals forces and hydrophobic interactions, the exposed bitter hydrophobic side chains on the peptide chain would spontaneously insert into the cyclic structure of cyclodextrin, reducing the surface hydrophobicity (from 43.76% to 30–37%). At the same time, as the pH value increased, the solubility, foaming, and emulsifying properties were enhanced. In the bitter flavor strategy, α-cyclodextrin encapsulation effectively retained the antioxidant activity and improved the sensory and physicochemical properties of the hydrolysate, highlighting the potential pathways for its development as a functional food component.

Furthermore, to address the core issue of insufficient strength of traditional alginate gels in digestive fluids, modern gel technology has introduced enhanced covalent or non-covalent cross-linking methods. Due to the ability to precisely control the cross-linking density between polysaccharide matrix and proteins, a three-dimensional gel network with controllable pore size can be constructed [76]. Such networks may reduce the oral release and diffusion of bitter peptides, thereby decreasing their opportunity to interact with TAS2R receptors. Their ability to preserve peptide conformation and bioactivity, however, should be verified for each specific peptide system.

#### 3.2.2. Emulsion- and Nanovesicle-Based Encapsulation

In multiphase liquid systems, constructing multi-level interface barriers through emulsions or liposomes can significantly alter the distribution kinetics of bitter peptides between the water/oil phases, effectively blocking the contact between the exposed hydrophobic groups of the bitter peptides and the TAS2R family receptors in the oral cavity. For instance, Gao et al. [77] demonstrated that in water-in-oil high internal phase emulsions (HIPEs) and water-oil-water (W1/O/W2) dual emulsions, precisely positioning the bitter peptides in the inner water phase (W1) can create a robust physical isolation barrier, where the dense oil phase (O) and the external continuous phase (W2) work together to completely cut off the release pathway of the bitter molecules in the oral environment. In such systems, the oil phase and gel network mainly function as oral barriers that reduce the immediate diffusion of bitter peptides toward taste receptors. After swallowing, digestion of lipids and biopolymers can gradually weaken the interfacial barrier, enabling delayed or controlled peptide release in the gastrointestinal tract.

In the application of liposomes, Ma et al. [78] developed a new type of phospholipid-based nanovesicles (PBNs), which form a nanoscale bilayer structure through the self-assembly of amphiphilic phospholipid molecules. This enables the encapsulation of bitter substances and reduces the intensity of bitterness to 1/12 of the original level. Additionally, by combining the W/O emulsion transfer method to construct a multilayer membrane nano-capsule structure, the thermodynamic stability of the interface has been significantly enhanced. Moreover, adding γ-cyclodextrin to PBNs enhances the adsorption of bitter substances to phospholipids and achieves good sustained release. This reduces bitterness and astringency. It mainly promotes the core advantages of the encapsulation method in terms of bitterness masking and overall delivery efficiency.

#### 3.2.3. Computation-Driven Prediction of Wall Materials and Cross Linking

The deep integration of artificial intelligence (AI) and advanced modeling techniques has significantly enhanced our understanding of the mechanism of bitterness formation through precise masking and encapsulation strategies. Based on this background, Cui et al. [65] innovatively constructed a machine learning composite prediction model that integrates molecular descriptors, molecular simulation, and integrated docking technology. This research successfully revealed the conserved binding sites of the human broad-spectrum bitter receptor TAS2R14 and its ligand recognition mechanism. At the atomic level, it precisely located the key amino acid residues that determine the binding affinity. Although such static docking models make it difficult to fully replicate the large molecule crowding effect of peptides in real food matrices, the receptor targeted recognition model established by them provides extremely important precise target information for subsequent physical embedding and debittering. Once the exposed specific hydrophobic residues in the peptide sequence that bind to the receptor are accurately locked by this computational model, future efforts can abandon the traditional blind encapsulation method and instead carry out targeted encapsulation design based on spatial structure. For example, functional wall materials such as β-cyclodextrin with specific cavity structures can be introduced to precisely match the bitter target points calculated by the model. By virtue of van der Waals forces and hydrophobic interactions, the hydrophobic cavity of β-cyclodextrin can preferentially and selectively engulf and mask these high bitter side chains marked by the model. From the AI prediction of targets to the precise masking by β-cyclodextrin, this linkage strategy not only achieves efficient spatial steric debittering at the molecular receptor level but also maximally retains the biological activity of the remaining conformations of the peptide, pointing out a promising direction for the industrial targeted innovation of functional low bitter peptides.

**Figure 3:**
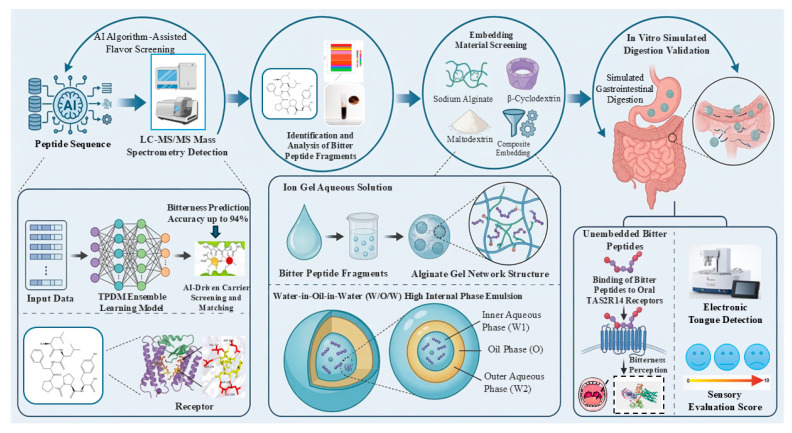
The application of AI-assisted encapsulation technology.

Figure 3 comprehensively and systematically demonstrates how AI, relying on machine learning models and computing tools, can rapidly predict the physicochemical properties, bitter-causing mechanisms, and application potential of peptides, thereby assisting in the design of peptide sequences with low bitterness characteristics [79,80]. At the same time, the introduction of AI technology also strongly supports the further development of low-cost, continuous encapsulation processes and effectively optimizes the stability of the encapsulation system in complex food processing environments, thus successfully adapting to the actual needs of large-scale production in the food industry.

### 3.3. In Silico Hydrolysis-Guided Enzymatic Debittering

The core of enzymatic debittering lies in the controlled use of specific proteases or peptidases, particularly endopeptidases and exopeptidases, to remodel the binding units (BUs) and stimulating units (SUs) of bitter peptides. Endopeptidases cleave internal peptide bonds and may either disrupt or generate bitter motifs depending on enzyme specificity, whereas exopeptidases, including aminopeptidases and carboxypeptidases, remove terminal residues from the N- or C-terminus [81]. Since many bitter peptides contain exposed hydrophobic or basic residues at terminal positions, selective terminal trimming can weaken their receptor-recognition motifs and reduce their interaction with TAS2R receptors. Nevertheless, the endpoint of enzymatic hydrolysis must be carefully controlled because excessive cleavage may reduce functional peptide sequences or generate new bitter fragments. Therefore, in silico hydrolysis-guided debittering should focus on identifying cleavage sites that disrupt bitter motifs while retaining bioactive domains.

The integration of modern enzymatic debittering with deep learning prediction models has shifted the process from empirical hydrolysis toward targeted cleavage design. The intervention of computational simulation fundamentally changes the process logic of enzymatic debittering. By using protein language models, molecular docking, and virtual hydrolysis tools, researchers can simulate the specific cutting patterns of proteases on a computer and predict the bitterness or functional potential of the generated peptide segments. Precise target confirmation of the core bitter sites rich in hydrophobic amino acids can be achieved, and combined with the computer simulation of the dynamic hydrolysis trajectories of different proteases, a high-throughput virtual peptideomics map can be generated [82]. Consequently, research shows that virtual peptidomics reshapes debittering logic. The study by He et al. [27] used the BIOPEP-UWM database to perform computer virtual enzymatic hydrolysis on wheat proteins and then conducted molecular docking with the bitter receptor TAS2R14. The study showed that although using papain to hydrolyze proteins into bioactive peptides can effectively reduce the content of gliadin and thereby weaken sensitization and enhance the safety of peptides, this hydrolysis process usually causes undesirable bitterness. To solve this problem, an AI-driven automated feature extraction technology precisely identifies the core bitter segments rich in hydrophobic amino acids located at the peptide chain ends or exposed surfaces from a large number of virtual fragments, while ingeniously avoiding known active functional motifs. By recommending the optimal combination of composite enzymes and enzyme source ratios in reverse, this system significantly reduces the overall bitterness of the system while retaining the maximum antioxidant activity of the peptides.

Therefore, Zhang et al. [83] utilized computational screening combined with sensory validation to discover that specific peptides derived from beef protein act as potent blockers for the bitter taste receptor T2R4. This strategy demonstrated that targeted hydrolysis can not only minimize the generation of bitter peptides but also yield peptides capable of competitively binding to bitter receptors. This directly blocks the transmission of bitter signals at the receptor level, effectively suppressing bitterness perception and enhancing the overall flavor profile of the system, while reducing the risk of losing peptide bioactivity.

#### Plastein Reaction

The plastein reaction, as a typical protease-catalyzed modification technique, is often used for the posttreatment of protein hydrolysis products. This reaction induces the polymerization of polypeptides, reducing the number of free amino groups while altering the structure of the peptide chain, thereby effectively masking the bitter peptides. Studies have shown that encapsulating 1% of the plastein reaction product in liposomes can significantly delay the release of the polypeptide, reduce the intensity of the bitter taste, and optimize the sensory quality [84]. Qian et al. [85] confirmed in research on soybean and whey protein hydrolysis products that through enzymatic peptide chain recombination, small molecule bitter peptides can aggregate into high-molecular-weight complexes; during this process, hydrophobic residues are effectively isolated within the complex, reducing the bitterness while enhancing the functional properties of the product.

From the perspective of molecular mechanisms, the plastein reaction mainly relies on the reverse condensation or transpeptidation catalyzed by proteases to recombine and polymerize small molecule bitter peptides with exposed hydrophobic groups into large molecular complexes. Since the bitter-causing residues are physically encapsulated within the large molecular structure, their ability to bind to taste receptor sites is lost in terms of spatial conformation, and they can also synergistically enhance the functional properties of plant protein hydrolysates [86]. Therefore, the plastein reaction can serve as an efficient enzymatic debittering strategy, achieving an overall improvement in the flavor of the system through structural modification and polymerization.

By further integrating deep learning prediction models, researchers can conduct in-depth analysis of the hydrolysis characteristics of different proteases to achieve precise enzyme selection and effectively predict the tendency of proteinases to produce bitter peptides. This intelligent enzymatic strategy, which combines targeted cleavage with reverse assembly, provides a new technical approach for simultaneously improving the flavor quality and biological activity of peptides, and realizes the coordinated optimization of food sensory characteristics and functional value.

### 3.4. Intelligent Optimization of Microbial Fermentation Parameters

Microbial fermentation to remove bitterness is essentially a complex metabolic network regulation process. Relying on the metabolic control of microorganisms, the extracellular enzyme system can target and cut the bitter precursor substances, converting them into molecules with lower sensory intensity of bitterness, thereby reducing the overall bitterness level of the system [87]. Traditional process screening often relies on manual experience or orthogonal experiments, which are not only inefficient but also prone to getting stuck in local optimal solutions. In view of this, recent research in fermentation engineering has begun to deeply integrate machine learning algorithms such as support vector machines (SVMs), random forests (RF), and response surface methodology (RSM) for the joint application, aiming to conduct high-dimensional global optimization of the entire fermentation process.

The research conducted by Han et al. [88] demonstrated that the low-salt fermented fish sauce system inoculated with Tetragenococcus halophilus 2MH-3 exhibited remarkable complexity in terms of quality. Using nano-LC-MS/MS proteomics technology, the researchers identified 10 types of umami peptides and 50 types of bitter peptides from the fermentation products. By constructing a cooccurrence correlation network between microorganisms and flavor peptides, the researchers clarified the key metabolic roles of core genera such as Tetragenococcus, Lactobacillus, and Staphylococcus in the generation of flavor peptides. Further, the researchers constructed the three-dimensional structures of the umami receptor T1R1/T1R3 and the bitter receptor TAS2R14 through homology modeling and confirmed the optimal binding stability through molecular docking and 50 ns molecular dynamics (MD) simulations. The results showed that the umami peptide U10 and the bitter peptide B68 had the highest flavor intensity. Notably, U10 could competitively bind to the active pocket of the TAS2R14 receptor, thereby blocking the interaction between the bitter peptide and the receptor and the subsequent activation of the signaling pathway. This mechanism of inhibiting the generation of bitter signals at the molecular transduction level effectively masked the perception of bitterness. The above molecular mechanisms confirmed that by enhancing the umami quality of low-salt fermented fish sauce, competitive binding-mediated debittering and enhancement of flavor can be achieved simultaneously.

During the fermentation process, the metabolic activities of microorganisms alter the chemical composition of the food matrix. For instance, they can mask bitterness by reducing the accumulation of bitter amino acids or increasing the concentration of sweet and savory compounds [89,90]. In the multistrain mixed fermentation system, the synergistic metabolism of microbial communities can effectively promote the degradation of bitter precursor proteins [91]. Additionally, researchers have integrated historical multiomics data covering different proportions of core strains, dynamic pH, dissolved oxygen (DO) levels, cycles, and temperatures into predictive models with strong analytical capabilities. Leveraging AI to deeply decode the complexity of fermentation phenotypes, the research can precisely locate the optimal metabolic balance point. It can simultaneously inhibit the formation of bitter peptide precursors to the greatest extent while promoting the efficient degradation of residual hydrophobic residues by specific microbial peptidases. The metabolic flux analysis (MFA) model based on transcriptomics and metabolomics data reveals the influence of fermentation stress environments on amino acid degradation pathways through network topological diagrams. This not only accelerates the dissipation of bitter molecules but also guides the carbon and nitrogen metabolism towards free amino acids and small peptides with savory or sweet flavors, from a targeted perspective. This targeted guidance supports the self-repair of the flavor of the fermentation system and the construction of the target profile [92,93], fully demonstrating the core value of the integrated approach in fermentation optimization.

Since microbial fermentation technology can also be used for deamidation or specific hydrolysis processes, the exposure of hydrophobic residues can be effectively reduced. However, there are still many challenges in the key fermentation processes, such as the loss of some bioactive peptides, limited yield, metabolic feedback inhibition, and the complexity of process regulation. Utilizing neural network models to optimize the fermentation process, combined with AI-assisted parameter control, shows great potential in reducing the generation of bitter substances or promoting their degradation. Through the optimization of fermentation process parameters, strain selection guidance, and bitter substance prediction evaluation, the application of microbial fermentation in the debittering of peptides has been significantly improved. By synergistically optimizing pH, time, and strain ratio using neural network models, it is possible to more clearly identify the optimal metabolic balance point between inhibiting the formation of bitter peptides and promoting their degradation, thereby directly converting these key variables into the best product quality.

## 4. Bitterness Evaluation Methods for Bitter Peptides

Bitterness quantification has long relied on sensory threshold concepts, and the bitterness threshold remains a key parameter for characterizing bitter peptides. Since then, the evaluation system has been continuously improved, and a core measurement standard based on the bitter taste threshold has been established. That is, the lowest concentration of the compound at which 50% of the subjects can clearly perceive the bitter taste is the minimum concentration, and this indicator is still the key quantitative parameter for characterizing the sensory properties of bitter peptides to this day [15]. Under the current AI and omics models, the evaluation system has gone beyond the traditional process verification function and has become the main source of high-fidelity training data for deep learning models. Currently, in the food field, a three-dimensional cross-validation framework integrating standardized sensory quantitative assessment and biomimetic electronic tongue technology has been established. While achieving precise and objective quantification of the intensity of bitter peptides, it also provides stable data support for the iterative optimization of AI models.

**Figure 4:**
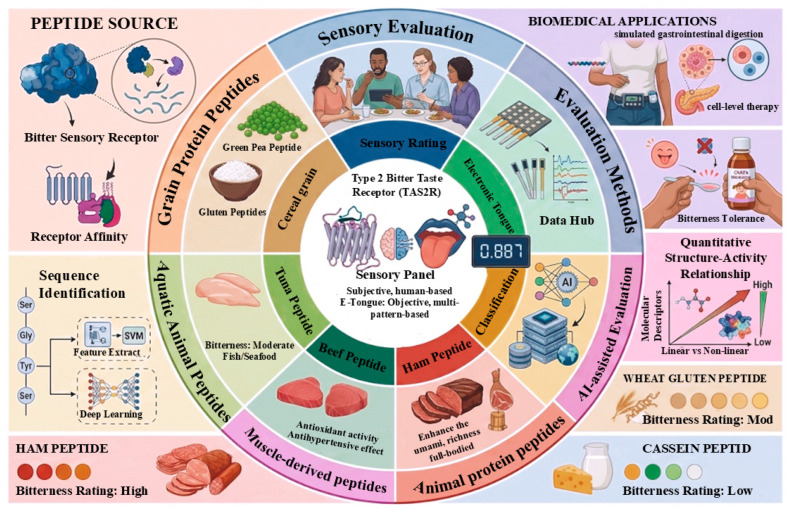
Multi-dimensional cross-validation evaluation system for bitter peptides.

Figure 4 presents the overall logical framework of the research on bitter peptides, covering the entire process from source identification and sensory evaluation to biological applications. This figure further elaborates on the integrated application of artificial intelligence and biomimetic technology in the precise quantitative determination of bitterness and the characterization of the structure–activity relationship.

### 4.1. Quantitative Sensory Evaluation System

Apart from structural factors, sensory evaluation remains the only means to directly reflect the true eating experience of bitter peptides in food quality through the human taste system. As shown in the figure, a multi-dimensional cross-validation evaluation system for bitter peptides has been established at present. Among them, quantitative descriptive analysis (QDA) holds a dominant position in both industrial applications and academic research of bitter peptide sensory evaluation. This method converts subjective taste perception into objective quantitative data through standardized experimental design. The physiological basis of this attribute transformation lies in the fact that after bitter peptides specifically bind to the bitter taste receptors on the tongue surface, they trigger an intracellular signal cascade amplification reaction, ultimately resulting in the perception of bitterness [94]. Based on this physiological mechanism, QDA can effectively quantify the intensity of bitterness and demonstrates high applicability in the verification and screening of bitter peptides [95].

Cosson et al. [96] employed the QDA method to conduct taste sensory scoring for pea protein isolates with different degrees of hydrolysis. They selected pre-trained evaluators and used a standard bitter solution of 1.5–4.5 mM caffeine, corresponding to a bitter score of 5–15 points. They conducted repeated intensive training to establish a stable memory of bitter intensity and enable the evaluators to master the standard system proficiently. Subsequently, the evaluators independently evaluated the enzymatic hydrolysate samples using an intensity scale. The final mean score was ultimately used as sensory data for subsequent modeling analysis. The data stability and method repeatability were also verified through retesting, fully confirming the reliability of the QDA process. Therefore, the reliability of QDA still depends on the selection, training, and performance monitoring of the assessors. In the evaluation of bitterness, standard bitter compounds such as caffeine or quinine should be used to pre-screen the evaluators for their sensitivity to bitterness, and only those with stable recognition thresholds and good discrimination abilities should be retained to participate in the training of the assessors. During the training process, reference solutions with bitterness intensity grading should be used to establish a unified intensity standard, and the samples should be prepared and presented under controlled, random, and blind conditions [97].

However, since this method mainly relies on the individual perception ability of the assessors, it inevitably leads to significant differences in tolerance and bitterness perception thresholds among different individuals. To overcome this defect, the current cutting-edge trend is to combine biomimetic sensing devices with proteomics technology for sensory evaluation. This multi-dimensional cross-validation strategy not only effectively compensates for the individual differences errors of artificial senses but also significantly improves the overall accuracy of bitterness data recognition and intensity quantification.

### 4.2. Biomimetic Electronic Tongue Sensor Evaluation

Given the inherent limitations of traditional manual sensory evaluation, such as low throughput and strong subjectivity, electronic tongue (E-tongue) systems have become useful complementary tools for bitterness screening. Unlike human assessors, E-tongues do not measure hedonic liking or emotional acceptance. Instead, they generate reproducible physicochemical response patterns from sensor arrays and chemometric models, which can be calibrated against human sensory data. Therefore, E-tongue analysis is particularly suitable for high-throughput ranking of bitter peptide fractions, monitoring debittering processes, and selecting representative samples for subsequent QDA or consumer validation.

For instance, Khan et al. [98] demonstrated that a biomimetic electronic tongue can detect bitter and astringent taste responses by monitoring current changes. The bitter substances can form hydrogen bonds and ionic bonds with chitosan and electrolytes in the gel matrix, thereby generating quantifiable electrical signals. Thanks to this mechanism, the electronic tongue has successfully achieved quantitative analysis of bitterness in real and complex samples and has been widely applied in the evaluation of bitterness in food systems including dairy protein hydrolysates, coffee, and tea [99]. A key question is whether E-tongue responses are consistent with trained sensory panel data. Newman et al. [100] compared an E-tongue with a trained sensory panel for assessing bitterness in dairy protein hydrolysates and reported strong correlations between the two approaches, with R^2^ = 0.98 for caffeine solutions and R^2^ = 0.94–0.99 for casein- and whey-based hydrolysates. These results indicate that E-tongue analysis can reduce the dependence on costly and time-consuming trained panels during preliminary bitterness screening. More recently, Nath et al. [101] applied an E-tongue to detect bitterness changes in milk-protein-derived peptides produced by enzymatic and microbial hydrolysis, further supporting its applicability to peptide bitterness monitoring. Nevertheless, E-tongue outputs should not be interpreted as direct consumer perception.

Although some studies have compared E-tongue responses with consumer sensory data, instrumental outputs should not be interpreted as direct consumer perception [102]. E-tongue systems can reduce assessor-related variability and improve screening efficiency, but they cannot eliminate all sensory bias or reproduce hedonic perception. Therefore, E-tongue analysis should be regarded as an efficient instrumental screening and standardization tool rather than a complete replacement for human sensory assessment.

### 4.3. Three-Dimensional Cross-Validation of Peptide Bitterness Intensity

The future development of the intelligent prediction framework urgently needs to rely on a high-quality data loop to break through the bottleneck of the model’s generalization ability. Existing studies have shown that the high-dimensional matrix data output by the electronic tongue can effectively compensate for the subjective defects of manual sensory evaluation. For example, the principal component analysis (PCA) response values obtained after dimensionality reduction can directly be used as the objective external validation set for deep learning models such as graph convolutional networks (GCNs) and BIPE [12].

Based on this preliminary foundation, Wei et al. [51] innovatively proposed a theoretical framework for a three-dimensional cross-validation system. This framework successfully achieved deep alignment and cross-validation of multi-dimensional data, including the objective electrical signals extracted by electronic tongues, the subjective scores from human sensory evaluations, and the receptor binding free energy predicted through molecular docking. This data loop strategy not only comprehensively enhanced the reliability and biological relevance of the quantification of bitterness intensity and the assessment of debittering processes but also profoundly demonstrated that the systematic transition from “microscopic theoretical prediction” to “macroscopic physical verification” has become the trend in the rational design of flavor peptide targets.

## 5. Conclusions

This paper systematically constructs a collaborative regulatory theoretical framework from intelligent prediction to targeted debittering and then to multi-dimensional evaluation, which holds significant guiding significance for development in this field. Deep learning models such as BitterPep-GCN, BIPE, and CPM-BP have successfully overcome the accuracy issues of traditional prediction methods. By integrating these models with peptideomics and molecular docking technologies, high-throughput and precise identification of bitter peptides in complex matrices and in-depth analysis of their molecular mechanisms can be achieved. However, the industrialization transformation in this field still faces many bottlenecks. The cross-matrix applicability of prediction models is insufficient, the compatibility of multi-process integration is poor, and the in vitro evaluation system cannot fully simulate real physiological digestion processes.

In response to these challenges, the future research focus should be on deeply integrating multi-source heterogeneous data derived from proteomics, sensoryomics, and molecular dynamics simulations and then constructing a standardized bitter peptide database with multiple matrices and sequence features. On this basis, a more universally applicable flavor and function integrated generative model that will strongly promote the targeted rational design of low bitterness and high activity peptides should be developed. Additionally, it is necessary to develop green, continuous debittering processes based on biocatalysis and natural macromolecular wall materials. Artificial intelligence technology should be utilized to conduct multi-objective global optimization of process parameters such as enzymolysis, fermentation, and encapsulation, thereby achieving the best balance between debittering efficiency, activity retention, and industrial production cost control. In terms of the evaluation system construction, combining the biomimetic oral–gastrointestinal digestion model with in vitro receptor activation experiments will help create a more complete bitter perception full-chain evaluation system. In summary, continuously deepening the integration and cross-innovation of these methodologies, achieving precise connection from in vitro prediction to in vivo efficacy, will not only provide comprehensive scientific support for the development of functional peptide products but also point out the direction for the future development of food flavor chemistry.

## Figures and Tables

**Table 1:** Bitterness characteristics and peptide-level roles of amino acid residues.

Amino Acid	Side-Chain Category	Free Amino Acid Bitterness	Role in Bitter Peptides
Trp (W)	Aromatic hydrophobic residue	One of the strongest bitter amino acids, showed the highest concentration–bitterness slope in recent sensory characterization	Aromatic side chain may strengthen hydrophobic and π-related interactions with TAS2Rs, high-risk residue when exposed or located near key receptor-binding motifs
Phe (F)	Aromatic hydrophobic residue	Strong bitter amino acid, highly potent in free amino acid tests	Phe-containing peptides often show strong bitterness, especially when Phe is terminal or exposed, aromaticity enhances receptor–pocket interactions
Tyr (Y)	Aromatic residue with both polar and hydrophobic features	Free Tyr is difficult to evaluate because of poor water solubility at taste-relevant concentrations	Tyr-containing peptides can contribute to bitterness, Phe/Tyr-rich peptides have been reported to show stronger bitterness
Ile (I)	Branched-chain hydrophobic residue	Strong bitter amino acid; among the more potent bitter amino acids	Provides a strong aliphatic hydrophobic surface, contributes to receptor–pocket insertion when terminally exposed
Leu (L)	Branched-chain hydrophobic residue	Bitter amino acid; relevant in free amino acid supplements	Leu-containing peptides are classical bitter peptide models. stronger bitterness is often observed when Leu is located at the C-terminus
Val (V)	Branched-chain hydrophobic residue	Bitter, but generally less potent than Trp, Phe, Ile, or Leu	Contributes to hydrophobic motifs, bitterness depends strongly on sequence context and terminal exposure
Met (M)	Sulfur-containing hydrophobic residue	Predominantly bitter, but lower potency than Trp, Phe, Ile, and Leu in concentration–response data	May contribute to hydrophobic bitter motifs, although usually less dominant than aromatic residues or BCAAs
Pro (P)	Cyclic imino acid with conformational restriction	Bitter and sweet; lower bitter potency as a free amino acid	Can bend peptide backbone through its imino ring and expose bitter motifs, important in many bitter peptides despite not being a simple hydrophobic driver
Arg (R)	Basic, positively charged residue	Clearly bitter, bitterness is less effectively suppressed by sodium salts than most hydrophobic bitter amino acids	May act as an electrostatic or stimulatory unit in bitter peptides, effect is receptor- and concentration-dependent
Lys (K)	Basic, positively charged residue	Bitter, salty, and savory, mixed taste profile with lower bitter potency	Can participate in electrostatic interactions, bitterness contribution is sequence-dependent
His (H)	Basic imidazole-containing residue; pH-sensitive	Bitter, salty, and savory, mixed taste profile	pH-sensitive residue; may alter receptor interaction or local charge environment
Ala (A)	Small aliphatic residue	Generally weak, not a dominant bitter amino acid	May slightly increase hydrophobicity in short peptides but is rarely a primary bitter driver alone
Gly (G)	Small flexible residue	Generally sweet, weak, not a major bitter amino acid	Increases peptide flexibility and may dilute hydrophobic density, can alter exposure of adjacent residues
Cys (C)	Sulfur-containing, weakly polar residue	Not a dominant bitter amino acid	Limited direct evidence as a major bitter residue, may affect oxidation, crosslinking, and matrix behavior
Ser (S)	Polar uncharged residue	Not a dominant bitter amino acid	Can participate in hydrogen bonding, usually reduces overall hydrophobicity
Thr (T)	Polar uncharged residue; essential amino acid	Not primarily bitter; noted as an exception among essential amino acids in recent sensory work	May dilute hydrophobicity and contribute hydrogen-bonding potential
Asn (N)	Polar uncharged residue	Not primarily bitter	Increases polarity and may reduce hydrophobic bitterness
Gln (Q)	Polar uncharged residue	Not primarily bitter	Increases polarity, may form hydrogen bonds and reduce hydrophobic character
Asp (D)	Acidic, negatively charged residue	Mainly sour, acidic rather than bitter	Acidic residues increase hydrophilicity and may weaken hydrophobic receptor binding
Glu (E)	Acidic, negatively charged residue; umami-related	Sour, umami rather than primary bitter	May reduce bitterness by increasing hydrophilicity, can contribute to umami or taste balance

**Table 2:** Comparison of key parameters of bitter peptides in prediction models.

Model Classification	Peptide Sequence and Bitterness Mechanism Resolution	Core Input Features	Advantages in Targeted Debittering Engineering	Ref.
Q-rule	Empirical judgment based on the average hydrophobicity (*Q* value) of peptide fragments (bitter if *Q* > 1400 cal/mol).	Amino acid composition ratios.	Rapid preliminary prediction of bitterness trends in crude extracts.	[15]
QSAR Models	Integration of physicochemical descriptors such as molecular weight, hydrophobicity, and amino acid sequence.	Chain length, sequence order, and terminal group characteristics.	Precise localization of bitter-inducing cores to guide targeted exopeptidase cleavage sites.	[18,21,30]
Peptidomics and Molecular Docking	High-throughput screening via LC-MS/MS coupled with TAS2R family receptor–ligand binding simulations.	Peptide sequences and 3D structures of TAS2R14 receptors.	Atomic-level elucidation of bitter mechanisms and highly efficient virtual screening.	[46,47,51]
BitterPep-GCN	Extraction of “bitter cores” formed by the clustering of aromatic residues after spatial folding.	3D spatial topological structures of peptides.	Precise capture of bitter-inducing motifs that are linearly distant but spatially proximal in conformation.	[11]
iBitter-GRE	Integration of ESM-2 protein language models with multilayer perceptrons for bitterness threshold regression in logarithmic space.	ESM-2 embeddings and multi-view sequence descriptors.	Improves bitter peptide prediction through stacked learning and multi-view feature fusion.	[57]
CPM-BP	Fusion of multi-dimensional sequence features with real-world cellular physiological responses.	Massive peptide sequences and corresponding sensory thresholds.	Directly quantifies and predicts bitterness thresholds, guiding virtual enzyme cleavage to avoid highly bitter sequences.	[59]

## Data Availability

No new data were created or analyzed in this study. Data sharing is not applicable to this article.
